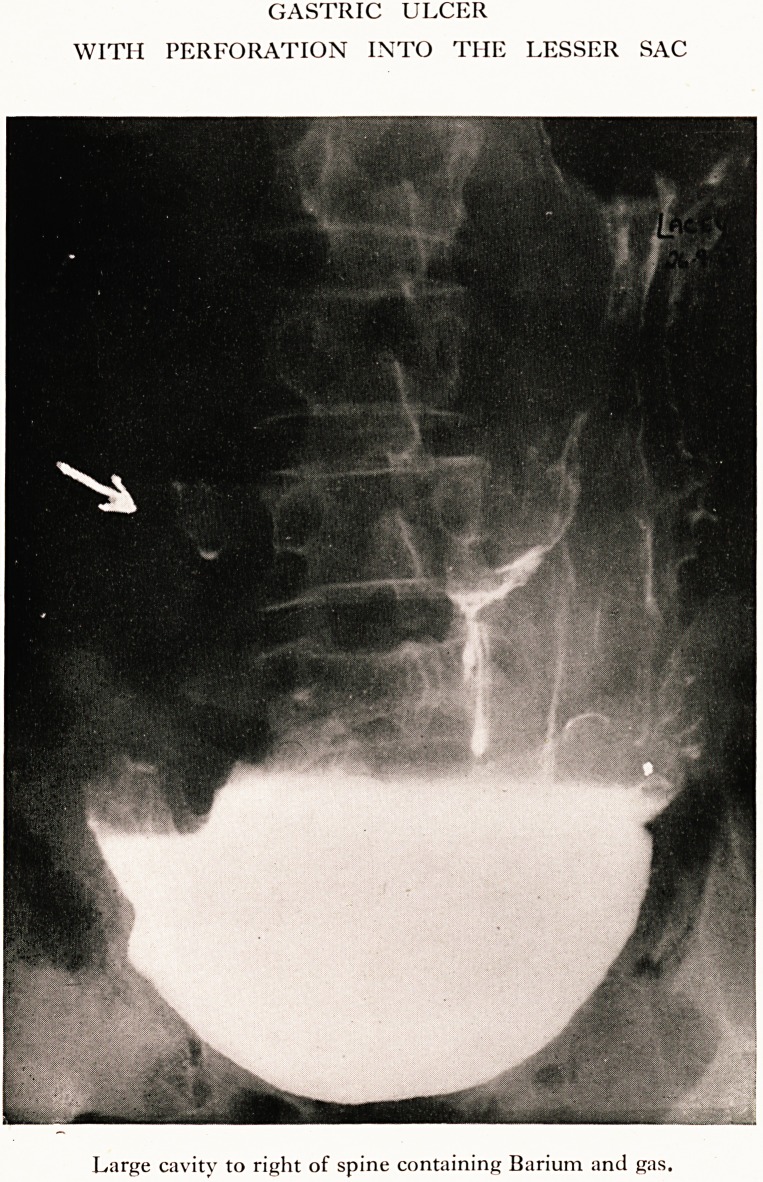# Gastric Ulcer with Perforation Into the Lesser Sac: *Report of a Case*

**Published:** 1950-04

**Authors:** J. A. Vere Nicoll

**Affiliations:** Surgeon, Yeovil District Hospital


### PLATE IV

### GASTRIC ULCER
### WITH PERFORATION INTO THE LESSER SAC

The ulcer crater seen above communicates with cavity shewn in Plate V.

### PLATE V

### GASTRIC ULCER
### WITH PERFORATION INTO THE LESSER SAC

Large cavity to right of spine containing Barium and gas.

### GASTRIC ULCER WITH PERFORATION INTO THE LESSER SAC:

### *Report of a case*

BY

J. A. VERE NICOLL, F.R.C.S.
*Surgeon, Yeovil District Hospital*

This case is considered to be of interest from the point of view of the length of history, the long latent period between the first symptoms and the onset of the acute phase, and the unusual size of the Haudec diverticulum which can be seen in the X-rays, with no history suggestive of an acute perforation at any time.

Mrs. L., aged sixty-seven, was admitted to Hospital complaining of severe epigastric pains and vomiting after all her meals. Her history was as follows:

She had upper abdominal pain associated with vomiting on and off throughout the 1914–18 war, which was put down to indigestion and treated with medicines. She was then free from pain until August 1949, when she started to have severe pain in the left of the epigastrium coming on half an hour after a meal, and being relieved slightly by medicines. The pain was the same type that she had previously experienced, though more severe in character. She was vomiting frequently and her general condition was poor.

Soon after admission she had a slight haematemesis and was put on a first stage ulcer diet—small feeds of citrated milk every two hours. Apart from epigastric tenderness there was no abnormality detected on physical examination. X-ray report: " There is a very large perforating ulcer halfway down the lesser curvature of a dilated and ptosed stomach, Plate IV; this apparently communicates with a cavity on the right side of the third lumbar vertebra which contains barium fluid and gas. The stomach is greatly dilated with much spasm of the pylorus." Plate V.

She was on dietetic treatment for twelve days and then had two small haematemeses followed by a severe one, and became rather collapsed. She was transfused after the severe haematemesis and was operated upon when her haemoglobin had risen to 78 per cent. It was found at operation that there was a large simple gastric ulcer with a stoma about an inch in diameter and adherent to the pancreas, leading into a false diverticulum which lay in the lesser sac. A sub-total gastrectomy was performed and the ulcer was sent for microscopical examination. The report stated that " sections show a chronic peptic ulcer with replacement of the mucous membrane by granulation tissue and underlying fibrosis. There is no evidence of malignancy."

The patient made an uneventful recovery and has remained well since. She was seen a few days ago and was enjoying her food and having no pain.

## Figures and Tables

**Figure f1:**
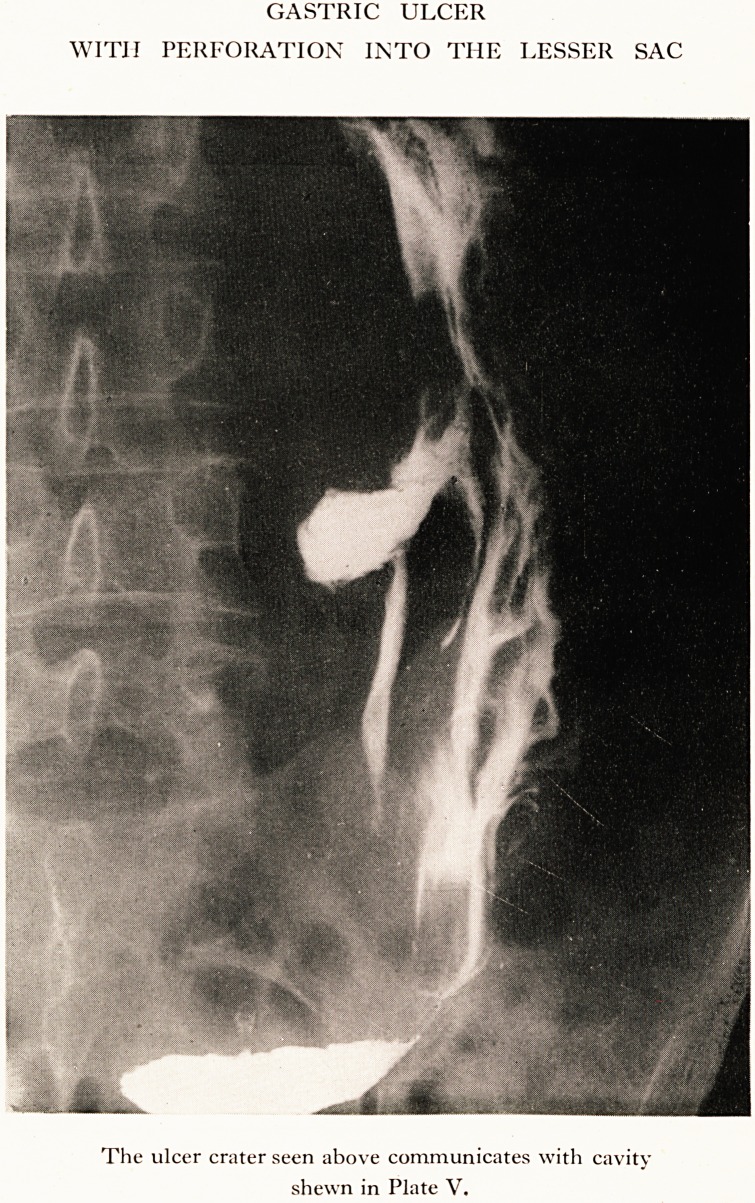


**Figure f2:**